# Marine Macrolides with Antibacterial and/or Antifungal Activity

**DOI:** 10.3390/md17040241

**Published:** 2019-04-23

**Authors:** Tomasz M. Karpiński

**Affiliations:** Department of Medical Microbiology, Poznań University of Medical Sciences, Wieniawskiego 3, 61-712 Poznań, Poland; tkarpin@ump.edu.pl or tkarpin@interia.pl; Tel.: +48-61-854-61-38

**Keywords:** macrolide, antibiotic, marine, antibacterial, antifungal, antimicrobial

## Abstract

Currently, the increasing resistance of microorganisms to antibiotics is a serious problem. Marine organisms are the source of thousands of substances, which also have antibacterial and antifungal effects. Among them, marine macrolides are significant. In this review, the antibacterial and/or antifungal activities of 34 groups of marine macrolides are presented. Exemplary groups are chalcomycins, curvulides, halichondramides, lobophorins, macrolactins, modiolides, scytophycins, spongistatins, or zearalanones. In the paper, 74 antibiotics or their analog sets, among which 29 with antifungal activity, 25 that are antibacterial, and 20 that are both antifungal and antibacterial are summarized. Also, 36 macrolides or their sets are produced by bacteria, 18 by fungi, ten by sponges, seven by algae, two by porifera, and one by nudibranch. Moreover, the chemical structures of representatives from each of the 34 groups of these antibiotics are presented. To summarize, marine organisms are rich in natural macrolides. Some of these may be used in the future in the treatment of bacterial and fungal infections. Marine macrolides can also be potential drugs applicable against pathogens resistant to currently known antibiotics.

## 1. Introduction

The marine world is rich in species and very diverse. Thus, marine organisms are a source of many substances with biological activity, including cytotoxic and antimicrobial. According to Burja et al., it was determined that in the marine world, there are over 13,000 unique compounds [1]. Important marine groups containing biologically active substances, including macrolides, are, among others, sponges [2] and cyanobacteria [3]. Swian et al. only described 121 compounds with antimicrobial activity among cyanobacteria, including the following chemical classes: alkaloids, aromatic compounds, pigments, fatty acids, phenols, macrolides, peptides, polyketides, porphinoids, and terpenoids [4]. Liu et al. described 118 marine macrolides, most with cytotoxic activity [5].

Macrolides are a group of polyketides. Currently, few of these substances are used in medicine. Among the antibacterial macrolides, the most important are erythromycin, azithromycin, roxithromycin, clarithromycin, josamycin, and spiramycin; among ketolides, telithromycin [6]. On the other hand, among antifungal polyene macrolides, amphotericin B, nystatin, and natamycin are most often used [7]. In general, antibacterial macrolides are active against *Staphylococcus* sp., *Streptococcus* sp., *Neisseria gonorrhoea*, *Haemophilus influenzae*, *Bordetella pertussis*, and *Neisseria meningitis*. Additionally, they are used in infections caused by intracellular pathogens, *Mycoplasma* sp. and *Chlamydia* sp. [8,9]. Clarithromycin is one of the antibiotics used in *Helicobacter pylori* infections [10]. The action of antibacterial macrolides is bacteriostatic. They reversibly bind to 23S ribosomal RNA of the 50s subunit of the bacterial ribosome inhibiting RNA-dependent protein synthesis [11]. The antifungal macrolides bind to ergosterol and lead to pore formation, leakage of monovalent ions (K^+^, Na^+^, H^+^ and Cl^−^), and finally to fungal cell death [12].

Recently, the increasing resistance of bacteria to antibiotics has become a serious problem. Globally, about 700,000 deaths every year may be caused by microorganisms resistant to antimicrobials [13]. In epidemiology, the most significant are the multidrug-resistant bacteria, e.g., *Escherichia coli*, *Klebsiella pneumoniae*, *Acinetobacter baumanii*, methicillin-resistant *Staphylococcus aureus* (MRSA), vancomycin-resistant MRSA, penicillin-resistant *Streptococcus pneumoniae* (PRSP), vancomycin-resistant *Enterococcus* (VRE), and extensively drug-resistant (XDR) *Mycobacterium tuberculosis* [14]. Antimicrobial resistance is increasingly common among both human and animal pathogens [10,15,16,17]. Antimicrobial resistance related to food containing zoonotic and fecal bacteria (*Salmonella* sp., *Campylobacter* sp., *Escherichia coli* and *Enterococcus* sp.) is also gaining importance [18]. The hope is to find new antibiotics to fight against multidrug-resistant strains. The source of these drugs could be marine macrolides.

In this paper, literature regarding the structures and biological (antibacterial and antifungal) activities of marine macrolides was examined. This literature was found by searching for articles published in PubMed/MEDLINE using combinations of the following keywords: “marine”, “macrolide/s”, “antibacterial”, “antifungal” and “antimicrobial”. Titles and abstracts of the resulting papers were examined to exclude or include articles for review. From the references of the included articles, additional works were selected. Finally, ninety-four papers have been incorporated into this narrative review.

The antibacterial and/or antifungal activities of 34 groups of marine macrolides are presented in this review. Moreover, the chemical structures of representatives from each group of these antibiotics are also represented. The origin and biological target of marine macrolides are presented in Table.

## 2. Antimicrobial Activity of Marine Macrolides

### 2.1. Macrolides 10-Membered 

#### 2.1.1. Curvulides

**Figure 1:**
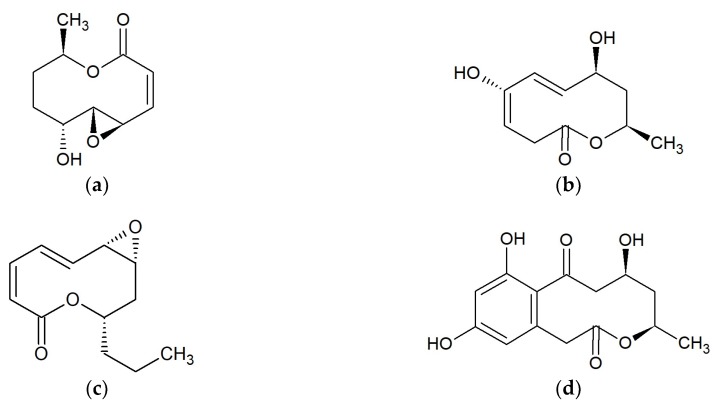
Chemical structures of 10-membered macrolides: (**a**) Curvulide A [19]; (**b**); Modiolide A [27]; (**c**) Phomolide A [28,29]; (**d**) Xestodecalactone B [33].

Curvulides are compounds obtained from strains of the fungus *Curvularia* sp. From one strain associated with the red alga *Acanthophora spicifera* occurring in Fingers Reef, Apra Harbor and Guam, 10-membered lactones have been isolated: curvulide A (Figure 1a [19]), curvulide B1 and B2 [20]. Curvularin and (S)-dehydrocurvularin obtained from *Curvularia* sp. strain M12, showed activity against fungus-like *Phytophthora capsici* exhibited in zoospore motility disorder [21]. Curvularin stereoisomers additionally possess anti-inflammatory activity [22] and are cytotoxic toward human tumor cell lines [23]. The two 11-hydroxycurvularin isomers isolated from the marine actinomycete *Pseudonocardia* sp. HS7 obtained from the sea cucumber *Holothuria moebii*, showed antibacterial activity towards *Escherichia coli* [24]. It was demonstrated that curvularin and αβ-dehydrocurvularin have anti-fungal activity against *Saccharomyces cerevisiae* (minimum inhibitory concentration (MIC) 375–750 μg/mL) and *Sclerotinia sclerotiorum* (MIC > 3000 μg/mL). Both substances also inhibited the growth of *Bacillus subtilis* (MICs of 1500 and > 3000 μg/mL), while αβ-dehydrocurvularin was additionally active against *Staphylococcus aureus* with an MIC of 375 μg/mL. Presented macrolides were not active against Gram-negative bacteria such as *Escherichia coli* and *Pseudomonas aeruginosa*. Both compounds were produced by the fungus *Eupenicillium* sp. associated with marine sponge *Axinella* sp. collected in the South China Sea near Sanya, China [25]. Curvulalide, curvulapyrone, and an uncyclized modiolide macrolide, curvulalic acid isolated from a sea fan-derived *Curvularia* sp. PSUF22 were not active against *Staphylococcus aureus* ATCC 25923, methicillin-resistant *S. aureus* SK1, or *Microsporum gypseum* SHMU-4 [26].

#### 2.1.2. Modiolides

To the 10-membered macrolides, belong modiolides A (Figure 1b) and B. Both are produced by fungus *Paraphaeosphaeria* sp. strain N-119, which was obtained from a marine horse mussel *Modiolus auriculatus* occurring in Hedo Cape, Japan. Modiolides A and B showed antibacterial activity against *Micrococcus luteus* (MIC = 16.7 mg/mL) and antifungal activity against *Neurospora crassa* (MIC = 33.3 mg/mL) [27]. Modiolide A is also the secondary metabolite of the marine-derived fungus *Curvularia* sp. Modiolide A and at least four substances resembling 10-membered lactones but featuring modified oxidation patterns around their macrocycles were shown to occur in this species [20]. In other studies, it was demonstrated that modiolide A obtained from *Curvularia* sp. strain M12, acts against the fungus-like eukaryotic microorganism *Phytophthora capsici,* leading to the disorder of zoospore motility at high concentrations (IC_50_: 50–100 μg/mL) [21]. Trisuvan et al. showed a lack of modiolide A activity against strains *Staphylococcus aureus* ATCC 25923, methicillin-resistant *S. aureus*, and *Microsporum gypseum* SH-MU-4 at the initial concentration of 200 μg/mL [26].

#### 2.1.3. Phomolides

Two 9-propyl-substituted 10-membered macrolides, phomolide A (Figure 1c) and B have been isolated from the marine fungus *Phomopsis* sp. hzla01-1. Both substances had significant activities against bacteria *Escherichia coli* CMCC44103, and fungi *Candida albicans* AS2.538 and *Saccharomyces cerevisiae* ATCC9763 with MIC values of 5–10 mg/mL [28,29]. Another similar chemical constituent, phomolide C, was obtained from the strain *Phomopsis* sp. B27 [30] and from the fungus *Diaporthe* sp., however it did not show antifungal activity against *Cochliobolus miyabeanus* [31].

#### 2.1.4. Xestodecalactones

Xestodecalactones A–C, were obtained from an isolate of the fungus *Penicillium* cf. *montanense* from the marine sponge *Xestospongia exigua* collected from the Bali Sea, Indonesia. Among these metabolites, xestodecalactone B was found to have anti-fungal activity against the yeast *Candida albicans* at concentrations of 20 µM and higher. Simultaneously, xestodecalactones A–C (Figure 1d) were inactive toward the bacteria *Bacillus subtilis*, *Staphylococcus aureus*, and *Escherichia coli* [32,33]. Xestodecalactones D–F obtained from *Corynespora cassiicola*, isolated from the Chinese mangrove plant *Laguncularia racemosa*, neither showed antibacterial nor antifungal activity [34].

### 2.2. Macrolides 12-Membered 

#### 2.2.1. Amphidinolides

**Figure 2:**
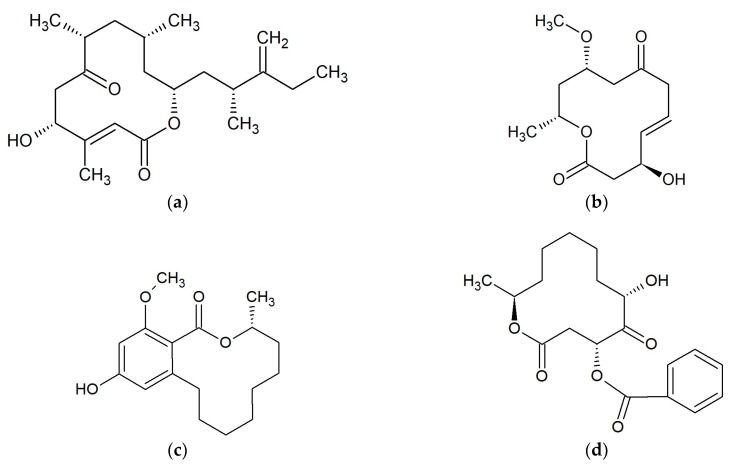
Chemical structures of 12-membered macrolides: (**a**) Amphidinolide Q [35]; (**b**) Dendrodolide A [36]; (**c**) Lasiodiplodin [37]; (**d**) Sporiolide A [40].

Amphidinolide Q (Figure 2a) and four analogs; amphidinins C–F, were isolated from the symbiotic dinoflagellate *Amphidinium* sp. The dinoflagellate *Amphidinium* sp. (2012-7-4A strain) was obtained from the marine acoel flatworm *Amphiscolops* sp. collected at Ishigaki Island, Okinawa, Japan. All compounds were active against *Trichophyton mentagrophytes* (MIC 16–32 μg/mL). Moreover, amphidinolide Q was active against *S. aureus*, *B. subtilis*, *Escherichia coli*, and *Candida albicans* (MICs 16–32 μg/mL) [35].

#### 2.2.2. Dendrodolides

Marine-derived *Cladosporium* fungi are a source of 12-membered macrolides dendrodolides A (Figure 2b), C, L, M and cladospolide B. *Cladosporium* sp. were cultivated from the gorgonian *Anthogorgia ochracea* obtained from the South China Sea. Three dendrodolides (A, C and M) showed antibacterial activity against *Bacillus cereus*, *Tetragenococcus halophilus*, *Staphylococcus epidermidis*, *Staphylococcus aureus*, *Escherichia coli*, *Pseudomonas putida*, *Nocardia brasiliensis*, and *Vibrio parahaemolyticus*, with MIC values ranging from 3.13 to 25.0 μM [36].

#### 2.2.3. Lasiodiplodins

Lasiodiplodins (Figure 2c) are resorcinolic macrolides [37] isolated among others from marine endophytic fungus No. ZZF36 connected with brown alga (*Sargassum* sp.). The fungus was collected from Zhanjiang Sea, China. Compound de-*O*-methyllasiodiplodin exhibited inhibitory activity against *Staphylococcus aureus* with an MIC of 6.25 µg/mL, and lower activities against *Bacillus subtilis*, *Salmonella enteritidis*, *Candida albicans* and *Fusarium oxysporum* f.sp. *cubense*. Lasiodiplodin inhibited the growth of *S. aureus*, *B. subtilis*, and *F. oxysporum* (MICs 25–100 µg/mL), while 5-hydroxy-de-*O*-methyllasiodiplodin was shown to be only effective against *S. aureus* at 100 µg/mL. Compound 6-oxo-de-*O*-methyllasiodiplodin was not active against all tested pathogens [38]. 

#### 2.2.4. Sporiolides

Sporiolides are 12-membered macrocyclic lactones. Sporiolides A (Figure 2d) and B were isolated from the fungus *Cladosporium* sp., which was separated from a marine brown alga *Actinotrichia fragilis* (Okinawa Island, Japan). Both sporiolides were active against *Micrococcus luteus* with a MIC value of 16.7 µg/mL. Moreover, sporiolide A showed antifungal activity against *Aspergillus niger*, *Candida albicans*, *Cryptococcus neoformans*, and *Neurospora crassa* with MICs of 8.4–16.7 µg/mL. Neither of the sporiolides were active against *Bacillus subtilis*, *Escherichia coli*, or *Paecilomyces variotii* [39,40]. 

### 2.3. Macrolides 14-Membered 

#### 2.3.1. Lobophorins

**Figure 3:**
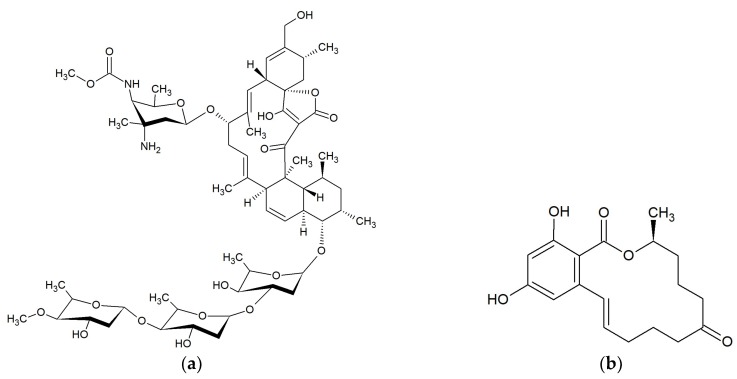
Chemical structures of 14-membered macrolides: (**a**) Lobophorin A [42]; (**b**) Zearalenone [46].

Lobophorins A (Figure 3a) and B were isolated from a marine Actinomycete strain #CNB-837 isolated from the surface of the Caribbean brown alga *Lobophora variegata* [41,42]. Spirotetronate antibiotics; lobophorins E and F, were isolated from *Streptomyces* sp. SCSIO 01127 obtained from sediment in the South China Sea [40]. Lobophorins H and I were obtained from *Streptomyces* sp. strain 12A35, which was isolated from the deep-sea sediment of the South China Sea [43]. Lobophorins A, B, E, and F exhibited activities against *Bacillus thuringensis* SCSIO BT01 with MIC values of 2–8 µg/mL. Lobophorin F displayed antibacterial activities against *Staphylococcus aureus* ATCC 29213 and *Enterococcus faecalis* ATCC 29212 with MIC values of 8 µg/mL [43]. Additionally, lobophorins B, F, I and H exhibited inhibitory activities against *Bacillus subtilis* CMCC63501. Lobophorins B and H showed strong activities (MICs of 1.57–3.13 μg/mL), while lobophorins F and I possessed moderate activities (MICs 6.25–50 μg/mL). Lobophorins F and H also had moderate activities against *Staphylococcus aureus* ATCC29213 with MIC values of 6.25–50 μg/mL. However, none of the studied compounds inhibited bacterium *Escherichia coli,* fungi *Candida albicans* or *Fusarium moniliforme* [44].

#### 2.3.2. Zearalanones

β-resorcylic acid lactones were obtained from the culture of a *Penicillium* sp. derived from cotton clothing drifting off Namhae Island, which were zearalanone analogs: 8’-hydroxyzearalanone, 2’-hydroxyzearalanol, zearalanone, β-zearalanol, zearalenone (Figure 3b), and β-zearalenol [45,46]. Some β-resorcylic macrolides were obtained from the marine *Fusarium* sp. O5ABR26 isolated from a sponge collected in the Miura Peninsula of Japan. Among these substances, zearalenone displayed the best inhibitory activity against fungus *Pyricularia oryzae* (MIC 6.25 μg/mL). Simultaneously, 8′-hydroxyzearalenone was less active with a MIC value of 200 µg/mL [47]. It was demonstrated that fungus *Fusarium* sp. PSU-ES73, isolated from the seagrass *Thalassia hemprichii* found throughout the shores of the Indian and the Western Pacific Oceans, contains β-resorcylic macrolides 5’-hydroxyzearalenone, zearalenone, 8’-hydroxyzearalenone, 7’-dehydrozearalenone, β-zearalenol, 5’-hydroxyzearalenol, and relgro. Only zearalenone exhibited weak activity against *Staphylococcus aureus* ATCC25923, methicillin-resistant *S. aureus* SK1 (MIC 400 μM), and *Cryptococcus neoformans* ATCC90113 (MIC 50.26 μM). The remaining compounds were inactive [48].

### 2.4. Macrolides 15- and 16-Membered 

#### Bromophycolides

**Figure 4:**
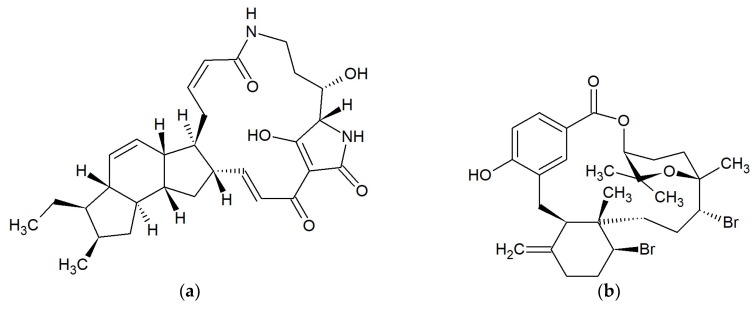

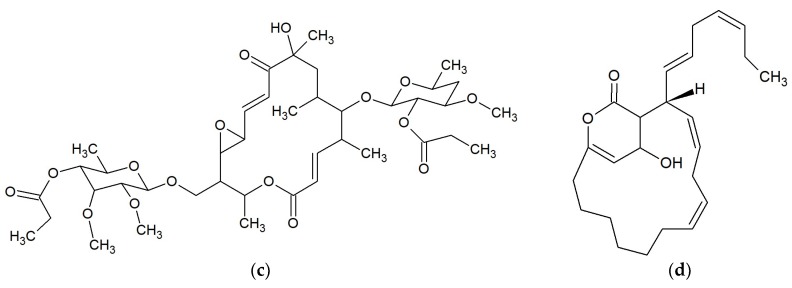
Chemical structures of 15- and 16-membered macrolides: (**a**) Butremycin [49]; (**b**) Bromophycolide P [50]; (**c**) Chalcomycin B [51]; (**d**) Neurymenolide A [53,54].

15- and 16-membered bromophycolides J-Q were isolated from extracts of the red alga *Callophycus serratus* from Yanuca, Fiji. Bromophycolides P (Figure 4b) and Q exhibited antibacterial activity against methicillin-resistant *Staphylococcus aureus* (MRSA) with an IC_50_ of 1.4 and 1.8 µM, respectively, and vancomycin-resistant *Enterococcus faecium* (VRE) with an IC_50_ of 13 and 5.8 µM, respectively [49].

### 2.5. Macrolides 16-Membered 

#### 2.5.1. Butremycin

Butremycin (Figure 4a) was isolated from *Micromonospora* sp. K310 obtained from mangrove river sediment in the Western Region of Ghana. Macrolide showed weak activity against *Staphylococcus aureus* ATCC 25923, *Escherichia coli* ATCC 25922 with a MIC of 50 µg/mL and some strains of methicillin-resistant *S. aureus* (MRSA) with a MIC > 50 µg/mL [50].

#### 2.5.2. Chalcomycins

Chalcomycin A and chalcomycin B (Figure 4c) were isolated from the marine strain *Streptomyces* sp. B7064 derived from mangrove sediment near Pohoiki, Hawaii (Pacific Ocean). Both compounds exhibited excellent activities against bacteria *Staphylococcus aureus* (MIC 0.39 µg/mL) and *Bacillus subtilis* (MIC 6.25 µg/mL), and low activities against *Escherichia coli* (MIC >50 µg/mL). Chalcomycins did not show any activity towards fungi *Candida albicans* or *Mucor miehei* [51]. Dihydrochalcomycin, chalcomycin and chalcomycin E were isolated from marine-derived *Streptomyces* sp. HK-2006-1. Two compounds, dihydrochalcomycin and chalcomycin, exhibited activities against *Staphylococcus aureus* with MICs of 4–32 µg/mL but were not active against *Escherichia coli*, *Candida albicans*, or *Aspergillus niger* [52]. 

#### 2.5.3. Neurymenolides

Two α-pyrone macrolides, neurymenolides A and B [53,54], were isolated from the red alga *Neurymenia fraxinifolia* collected from Taveuni, Fiji. Neurymenolide A (Figure 4d) possessed activity against methicillin-resistant *Staphylococcus aureus* with an IC_50_ of 2.1 μM and vancomycin-resistant *Enterococcus faecium* with an IC_50_ of 4.5 μM [53].

### 2.6. Macrolides 18-Membered 

#### 2.6.1. Borrelidins

**Figure 5:**
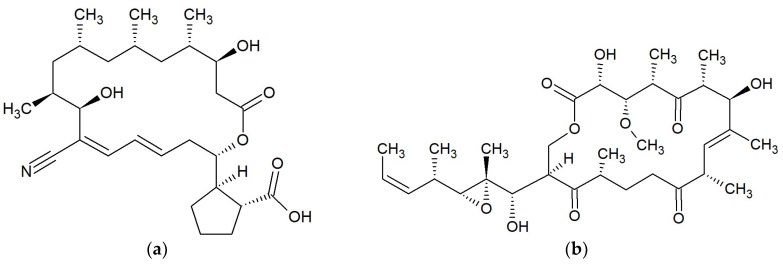

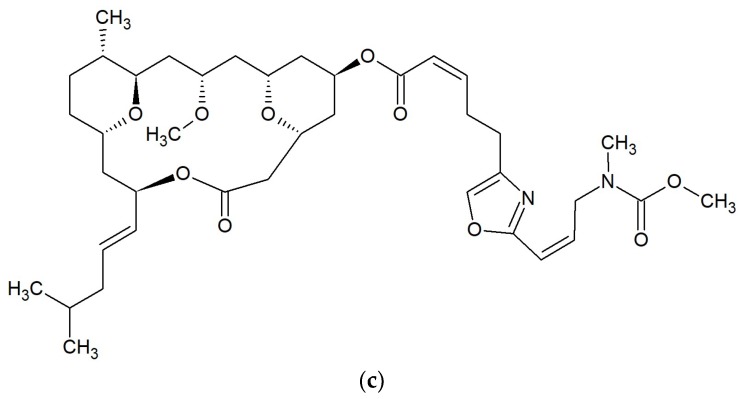
Chemical structures of 18-membered macrolides: (**a**) Borrelidin [55]; (**b**) 13-Deoxytedanolide [58,59]; (**c**) Leucascandrolide A [56].

Halophilic actinomycete *Nocardiopsis* sp. strain HYJ128 inhabiting a hypersaline saltern in Jeungdo, Jeollanam-do, Republic of Korea, produced 18-membered macrolides: borrelidin (Figure 5a) and borrelidins C–E. Borrelidin inhibited *Enterococcus faecalis* ATCC 19433, *E. faecium* ATCC 19434, *Proteus hauseri* NRBC 3851, *Klebsiella pneumoniae* ATCC 10031, and *Salmonella enterica* ATCC 14028 with MICs of 0.51–65 μM. Borrelidins C and D displayed inhibitory activity against *S. enterica* with MIC values of 16–63 μM. Borrelidin E did not exhibit any inhibitory activity against the tested bacteria [55].

#### 2.6.2. Leucascandrolides

Leucascandrolide A (Figure 5c) [56] was isolated from the calcareous sponge *Leucascandra caveolata*, collected along the east coast of the Coral Sea, New Caledonia. This compound strongly inhibited fungi *Fusarium oxysporum*, *Helminthosporium sativum*, *Phytophthora hevea*, *Botrytis cinerea*, *Pyricularia oryzae*, and yeast *Candida albicans* [57].

#### 2.6.3. Tedanolides

13-Deoxytedanolide (Figure 5b) is an 18-membered macrolide, which was isolated from the sponge *Mycale adhaerens* in Japan [58]. This macrolide strongly binds to the 60S large ribosomal subunit, causing inhibition of polypeptide elongation in fungus *Saccharomyces cerevisiae*, however it does not inhibit the polypeptide synthesis in bacterium *Escherichia coli* [59].

### 2.7. Macrolides 20-Membered 

#### 2.7.1. Macrocyclic Polyesters

**Figure 6:**
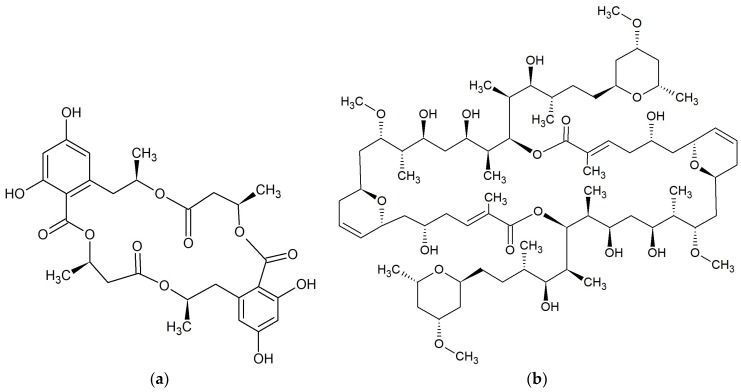
Chemical structures of 20-membered macrolides: (**a**) 15G256ι [61]; (**b**) Misakinolide A [62,63].

The marine fungus *Hypoxylon oceanicum* (strain LL-15G256) from mangrove wood collected in Shenzhen, China, produced macrocyclic polyesters [60]. A 20-membered compound 15G256ι (Figure 6a) exhibited low activity against the fungus *Neurospora crassa* acting as inhibitors of fungal cell wall formation. The 30-membered substance 15G256w had similar activity [61].

#### 2.7.2. Misakinolides

According to Sakai et al. misakinolide A (Figure 6b) is a 20-membered macrolide [62], however this macrolide occurs as 40-membered dimer [63]. Misakinolide A was isolated from the sponge *Theonella* sp., collected at Maeda-misaki, Okinawa, Japan. This compound possesses antifungal activity against *Candida albicans* (MIC 5 µg/mL) [62].

### 2.8. Macrolides 22-Membered 

#### 2.8.1. Kabiramides

**Figure 7:**
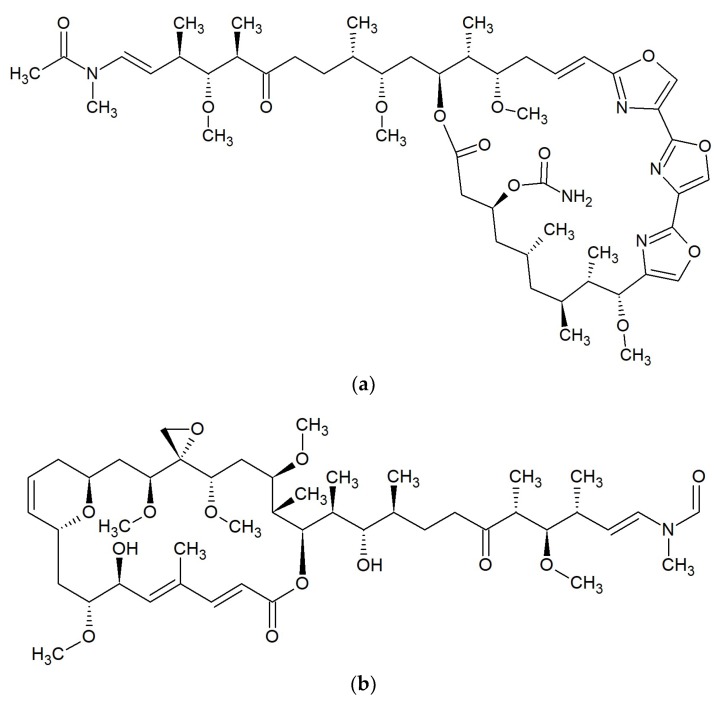
Chemical structures of 22-membered macrolides: (**a**) Kabiramide C [65]; (**b**) Tolytoxin [67].

Kabiramide C (Figure 7a) was isolated from the eggmasses of an unidentified nudibranch collected at Kabira Bay on Ishigaki-jima Island of the Ryukyus Islands, Japan. This 22-membered macrolide showed marked antifungal activity against *Candida albicans* ATCC 10234, *Aspergillus niger* ATCC 9642, *Penicillium citrium* ATCC 9849 and *Trichophyton interdigitale* [64]. Kabiramides G, J and K were isolated from the sponge *Pachastrissa nux* collected in the Gulf of Thailand. These macrolides, together with kabiramides B–D, showed anti-parasite activity against *Plasmodium falciparum* K1 [65].

#### 2.8.2. Scytophycins

Ishibashi et al. demonstrated that scytophycins A–E isolated from terrestrial blue-green alga *Scytonema pseudohofmanni* collected from Oahu, Hawaii, exhibited cytotoxicity and antifungal activity [66]. Scytophycin B, scytophycin E, 6-hydroxyscytophycin B, and tolytoxin (Figure 7b) (6-hydroxy-7-*O*-methylscytophycin B) obtained from the terrestrial blue-green alga *Cylindrospermum muscicola*, isolated on the island of Kauai, Hawaii also had antifungal activity [67]. Tolytoxin and two analogs; 6-hydroxyscytophycin B and 19-*O*-demethylscytophycin C were also produced by strains of *Scytonema mirabile*, *S. burmanicum*, and *S. ocellatum*. These macrolides had antifungal activity against *Aspergillus oryzae*, *Candida albicans*, *Penicillium notatum*, and *Saccharomyces cerevisiae* [68]. Tolytoxin isolated from blue-green alga *Tolypothrix conglutinata* var. *colorata* found at Fanning Island, Kiribati, exhibited additional inhibitory activity against *Alternaria alternata*, *Bipolaris incurvata*, *Calonectria critalarae*, *Colletotrichum coccodes*, *Phyllosticta capitalensis*, *Phytophthora nicotianae*, *Rhizoctonia solani*, *Sclerotium rofsii*, *Thielaviopsis paradoxa*, and *Trichophyton mentagrophytes* with MICs of 0.25–8 nM. Tolytoxin did not show any inhibitory activity against bacteria [69]. The presence of scytophycins with activity against *Candida albicans* and *Aspergillus flavus* has also been demonstrated in cyanobacteria *Anabaena* sp. HAN21/1, *Anabaena* cf. *cylindrica* PH133, *Scytonema* sp. HAN3/2, and *Nostoc* sp. HAN11/1 [70]. 

### 2.9. Macrolides 22–25-Membered 

#### 2.9.1. Gageomacrolactins

**Figure 8:**
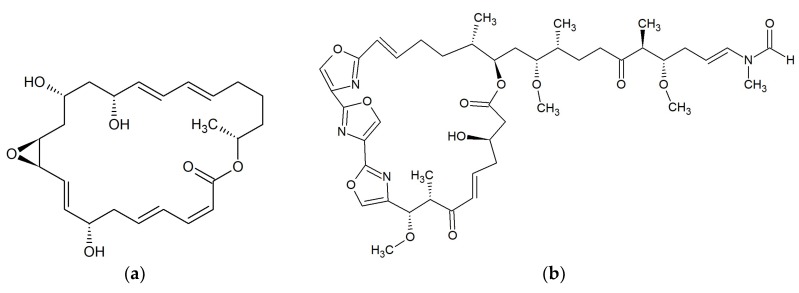

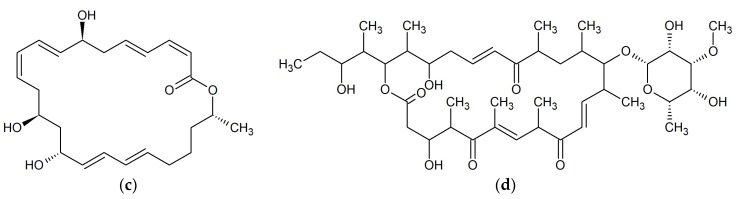
Chemical structures of 22–25-membered macrolides: (**a**) Gageomacrolactin 1 [71]; (**b**) Halichondramide [72]; (**c**) Macrolactin A [77,78]; (**d**) Maduralide [88].

*Bacillus subtilis* isolated from marine sediment collected from Gageocho, Republic of Korea, produced three gageomacrolactins (Figure 8a), which are 24-membered macrolactin derivatives. Gageomacrolactins displayed strong activity against some bacteria (*Staphylococcus aureus*, *Bacillus subtilis*, *B. cereus, Escherichia coli*, *Salmonella typhi*, and *Pseudomonas aeruginosa*) with MIC values of 0.02–0.05 μM. Additionally, isolated gageomacrolactins and macrolactins A, B, F, and W inhibit the growth of *Aspergillus niger*, *Botrytis cinerea*, *Colletotrichum acutatum*, *Candida albicans*, and *Rhizoctonia solani* with MIC values of 0.04–0.3 μM [71]. 

#### 2.9.2. Halichondramides

An oxazole-containing macrolide, halichondramide (Figure 8b), is a 25-membered antibiotic [72]. It was obtained from the sponge *Halichondria* sp. from Kwajalein Island, Marshall Islands, and showed significant activity against *Candida albicans* (MIC 0.2 pg/mL) and *Trichophyton mentagrophytes* (MIC 12.5 pg/mL). Halichondramide did not inhibit bacteria [73]. Further studies revealed that the sponge of the genus *Halichondria* sp. also contains two other macrolides (dihydrohalichondramide and isohalichondramide) having significant activity against *C. albicans.* In this same paper, the authors showed that anti-*C. albicans* activity had the nudibranch *Hexabranchus sanguineus*, from which dihydrohalichondramide and tetrahydrohalichondramide were isolated [74]. From the marine sponge *Chondrosia corticata* collected from Guam, more oxazole-containing macrolides were isolated: neohalichondramide, (19Z)-halichondramide, and secohalichondramide. These compounds exhibited antifungal activity toward the *Candida albicans* and *Aspergillus niger* [75]. Chung et al. in the sponge *C. corticata* identified the following macrolides: halichondramide, jaspisamide A, halishigamide D, neohalichondramide, and (19Z)-halichondramide. None of the compounds were active against Gram-positive or Gram-negative bacteria at 100 µg/mL. Halichondramide showed inhibitory activity against *Candida albicans*, *Aspergillus fumigatus*, *Trichophyton rubrum*, and *T. mentagrophytes* with MIC values of 0.2 to 0.91 µM. Compound (19Z)-halichondramide showed inhibitory activity against all tested fungi with MIC values of 0.78 to 14.55 µM. In the presented study, jaspisamide A, halishigamide D, and neohalichondramide were inactive at 100 µg/mL [76]. 

#### 2.9.3. Macrolactins

Macrolactins are a big group of 22- to 25-membered lactone macrolides. Some of these were isolated from a culture of *Bacillus* sp. PP19-H3 obtained from the macroalga *Schizymenia dubyi* collected on the Omaezaki coast of Shizuoka prefecture in Japan. Macrolactins A (Figure 8c), F, G, I, J, K, and L are 24-membered macrolides, macrolactin H is 22-membered, and macrolactin is M a 25-membered lactone. Macrolactins A, G, H, I, J, L, and M were effective against *Staphylococcus aureus* (MICs 5–10 ppm), and *Bacillus subtilis* (MICs 30–60 ppm). The macrolactins F and K had low activity against the above bacteria (MICs 80 and >100). None of the studied macrolides inhibited *Escherichia coli* or *Salinivibrio costicola* [77,78]. In other studies, macrolactin A did not have any antimicrobial activity [79,80].

Macrolactins A, B, F, and W isolated from marine *Bacillus subtilis* from Gageocho, Republic of Korea, inhibit the growth of *Aspergillus niger*, *Botrytis cinerea*, *Colletotrichum acutatum*, *Candida albicans*, and *Rhizoctonia solani* with MIC values of 0.04–0.3 μM [71]. 

7-*O*-succinylmacrolactin A and 7-*O*-succinylmacrolactin F, together with macrolactin F, were isolated from the marine *Bacillus* sp. Sc026 occurring in sediments around Sichang Island, Thailand. These two succinylmacrolactins showed activity against *Bacillus subtilis* and *Staphylococcus aureus* [78]. 7-*O*-malonylmacrolactin A was isolated from soil *B. subtilis* from Takalar, South Sulawesi in Indonesia. This compound inhibited methicillin-sensitive *S. aureus* (MSSA), methicillin-resistant *S. aureus* (MRSA), vancomycin-resistant enterococci (VRE), *Burkholderia cepacia*, and *Candida crusei* [79,80].

Macrolactin N was obtained from *Bacillus subtilis* AT29 and had antibacterial activity against *Escherichia coli, Staphylococcus aureus*, and *Bacillus subtilis*. It inhibited the growth of *E. coli* with a MIC value of 100 µg/mL, while for *S. aureus* and *B. subtilis,* the MIC_50_ is 100 µg/mL. Macrolactin N inhibited *S. aureus* peptide deformylase with an IC_50_ value of 7.5 µM [81].

From the marine *Bacillus* sp. derived from the sea sediment of East China Sea, macrolactin S, a 24-membered ring lactone, was obtained. Macrolactin S, together with macrolactins A and B had antibacterial activity against *Escherichia coli*, and *Staphylococcus aureus* [82]. 

Macrolactins T and U, along with macrolactins A, B, D, O, and S, were isolated from the bacterium *Bacillus marinus*, which was separated from *Suaeda salsa* collected in the coastline of the Bohai Sea of China. In the study, authors reported the inhibitory activity of macrolactins B (MIC 4.5–20.1 µg/mL) and D (MIC > 100 µg/mL) against fungi *Pyricularia oryzae* and *Alternaria solani*, and bacterium *Staphylococcus aureus* [83].

From marine bacterium *B. amyloliquefaciens* SCSIO 00856 isolated from the South China Sea gorgonian *Junceella juncea,* macrolactin V and S. Macrolactin V were obtained and had strong antibacterial activities against *Escherichia coli, Bacillus subtilis,* and *Staphylococcus aureus* with a MIC value of 0.1 μg/mL. Macrolactin S showed potent activity against *E. coli* and *S. aureus* (MICs 0.1–0.3 μg/mL), and weak against *B. subtilis* (MIC 100 μg/mL) [84]. 

Macrolactin W was isolated from a marine *Bacillus* sp. 09ID194 collected from Ieodo, a southern reef of South Korea. This macrolide showed antibacterial activities towards *Bacillus subtilis*, *Staphylococcus aureus*, *Escherichia coli*, and *Pseudomonas aeruginosa* with a MIC of 64 µg/mL [85].

One of the marine *Bacillus* sp. produces three 24-membered macrolactins, which contain an oxetane, an epoxide, and a tetrahydropyran ring, respectively. All three macrolactins showed antimicrobial activity against *Bacillus subtilis* and *Escherichia coli* (MIC 0.16 μM). The macrolactin with an epoxide ring also had excellent activity against *Saccharomyces cerevisiae* (MIC 0.02–0.16 μM) [86]. 

From *Bacillus subtilis* MTCC 10403, isolated from the brown seaweed *Anthophycus longifolius* collected from the Gulf of Mannar of India, new antimicrobial aryl-crowned polyketide macrolactin was obtained. This substance had bactericidal properties against *Escherichia coli*, *Aeromonas hydrophilla*, *Pseudomonas aeruginosa*, and *Vibrio* sp. at a low concentration with MIC < 13 µg/mL, and against *Klebsiella pneumoniae* with MIC ~25 µg/mL. The mode of antimicrobial action of this new acryl-crowned macrolactin was found to be iron chelating similar to siderophores [87].

#### 2.9.4. Maduralide

Maduralide (Figure 8d) is 24-membered ring macrolide. It was isolated from an unidentified marine bacterium of the order Actinomycetales in the shallow waters of Bodega Bay, USA. Maduralide shows weak antibiotic activity against *Bacillus subtilis* [88].

### 2.10. Macrolides 26-Membered 

#### 2.10.1. Neomaclafungins

**Figure 9:**
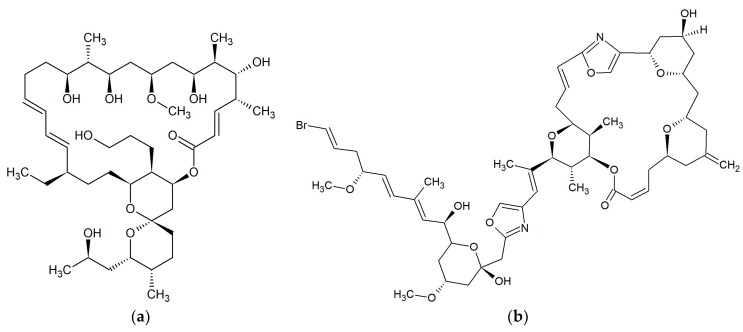
Chemical structures of 26-membered macrolides: (**a**) Neomaclafungin A [89]; (**b**) Phorboxazole A [90].

Neomaclafungins A–I (Figure 9a) were produced by the bacteria *Actinoalloteichus* sp. NPS702 isolated from the marine sediment of Usa Bay, Kochi Prefecture, Japan. These oligomycin macrolides exhibited significant antifungal activity in vitro against *Trichophyton mentagrophytes* ATCC 9533, with MIC values of 1–3 µg/mL [89]. 

#### 2.10.2. Phorboxazoles

Phorboxazoles A (Figure 9b) and B were isolated from the Indian Ocean marine sponge *Phorbas* sp. Both antibiotics had antifungal activity against *Candida albicans* and *Saccharomyces carlsbergensis*. None of these compounds showed any activity against *Escherichia coli*, *Pseudomonas aeruginosa*, or *Staphylococcus aureus* [90].

### 2.11. Macrolides 31-Membered 

#### Reedsmycins

**Figure 10:**
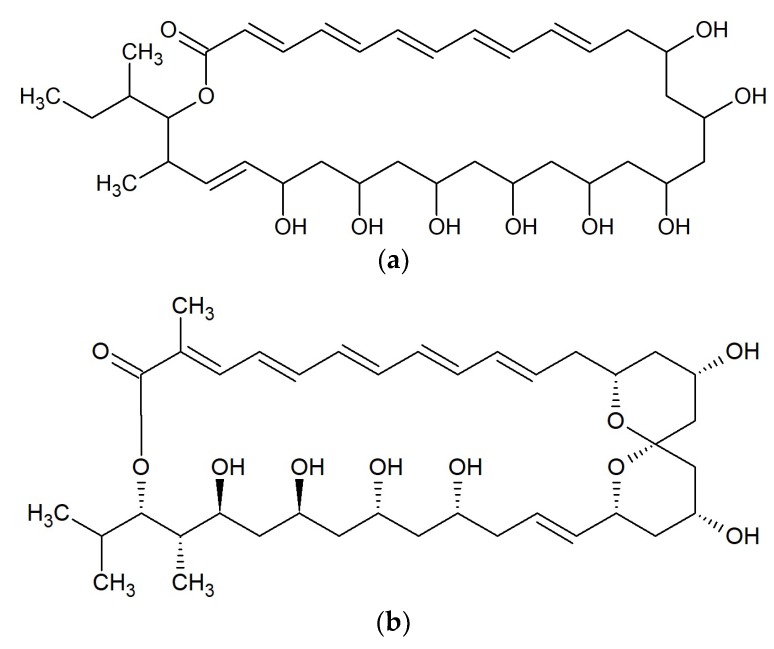
Chemical structures of 31-membered macrolide: (**a**) Reedsmycin A [92]; and 34-membered macrolide: (**b**) Marinisporolide A [93].

Reedsmycins are nonglycosylated polyene-polyol macrolides produced by marine-derived *Streptomyces youssoufiensis* OUC6819 [91] and by *Streptomyces* sp. CHQ-64 [92]. Reedsmycin A (Figure 10a) exhibited antifungal activity against *Candida albicans* (MIC 25–50 μM). Other compounds in this group had lower activity. MICs for reedsmycins C–E were 50–100 μM, for reedsmycin B were 100–200 μM. Reedsmycin F exhibited no inhibitory activity [92].

### 2.12. Macrolides 34-Membered 

#### Marinisporolides

Two polyene-polyol macrolides; marinisporolides A (Figure 10b) and B were isolated from the marine actinomycete *Marinispora* strain CNQ-140, collected offshore from La Jolla, California, USA. Both marinisporolides showed weak or no antifungal activity against *Candida albicans* with a MIC value of 22 μM [93]. 

### 2.13. Macrolides 36-Membered 

#### 2.13.1. Azalomycins

**Figure 11:**
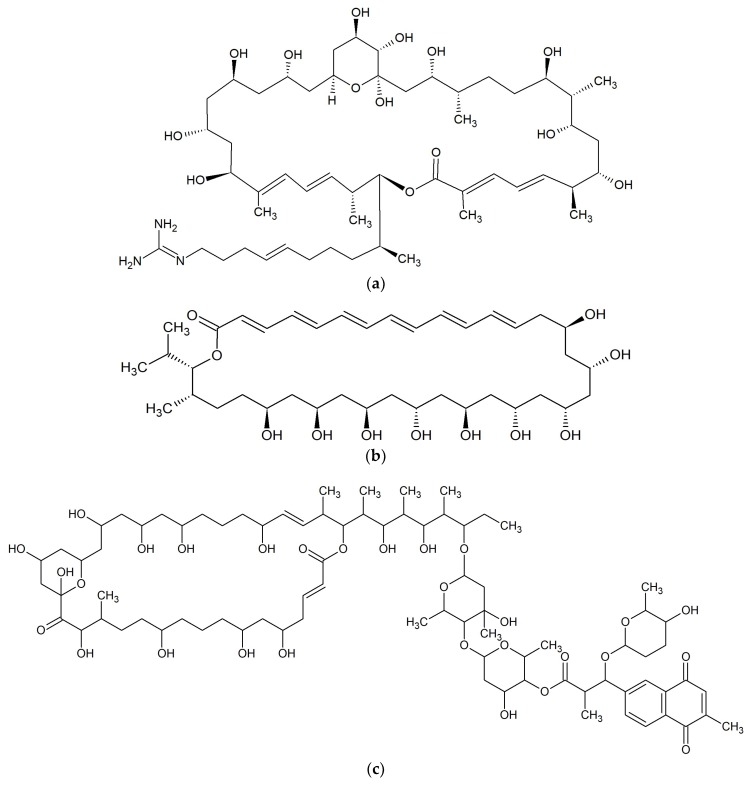

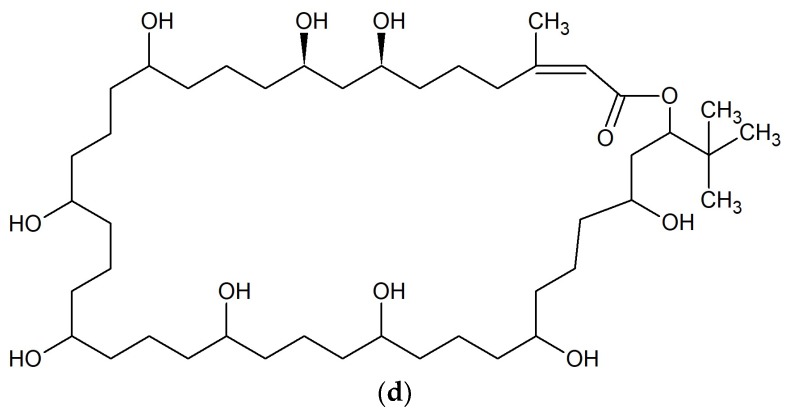
Chemical structures of 36-membered macrolides: (**a**) Azalomycin F [95]; (**b**) Bahamaolide A [96]; (**c**) PM100117 [97]; and 40-membered macrolide: (**d**) Amantelide A [99].

Two macrocyclic lactones, azalomycin F4a 2-ethylpentyl ester and azalomycin F5a 2-ethylpentyl ester, were identified from metabolites of *Streptomyces* sp. 211726 isolated from mangrove rhizosphere soil of *Heritiera globosa* collected in Wenchang, China. Both compounds showed moderate activity against *Candida albicans* ATCC 10231 at the MICs of 2.34 and 12.5 μg/mL [94]. Seven analogs of azalomycin F (Figure 11a) were identified from this strain with the same fermentation condition and showed antimicrobial activity against *C. albicans* ATCC 10231 (MICs 1.56–6.25 μg/mL), *Staphylococcus aureus* S014 (MICs 0.39–1.56 μg/mL), *Bacillus subtilis* S028 (MICs 0.20–0.78 μg/mL), and *Escherichia coli* S002 (MICs 3.13–25.00 μg/mL) [95].

#### 2.13.2. Bahamaolides

From the marine actinomycete *Streptomyces* sp. CNQ343 derived from sediment collected at North Cat Cay in the Bahamas, bahamaolides A and B. Bahamaolide A (Figure 11b) displaying significant inhibitory activity against *Candida albicans* ATCC 10231 with a MIC value of 12.5 μg/mL acting on enzyme isocitrate lyase were isolated. It also possessed antifungal activity against various pathogenic fungi: *Aspergillus fumigatus* HIC 6094, *Trichophyton rubrum* IFO 9185, *T. mentagrophytes* IFO4 0996. Bahamaolide B did not inhibit any tested strain [96].

#### 2.13.3. Polyhydroxyl Macrolides

Two polyhydroxyl macrolide lactones, PM100117 (Figure 11c) and PM100118 were isolated from the marine actinobacteria *Streptomyces caniferus* GUA-06-05-006A. Both substances possessed antifungal activity against *Candida albicans* ATCC10231 [97]. PM100117 also showed antibiotic activity against *Saccharomyces cerevisiae* W303.1A but was not active towards *Micrococcus luteus* [98].

### 2.14. Macrolides 40-Membered 

#### Amantelides

Amantelides A (Figure 11d) and B were isolated from gray cyanobacterium belonging to the family Oscillatoriales, collected near Puntan dos Amantes, Tumon Bay, Guam. The antifungal activity of amantelide A was observed against the marine fungi *Dendryphiella salina*, *Lindra thalassiae*, and *Fusarium* sp. at a concentration of 62.5 μg/mL. Moreover, macrolide had weak antibacterial activity against *Staphylococcus aureus* and *Pseudomonas aeruginosa* with a MIC of 32 μM. Amantelide B inhibited the growth of *Dendryphiella salina* at a concentration of 6.25 μg/mL [99].

### 2.15. Macrolides 42-Membered 

#### Spongistatins

**Figure 12:**
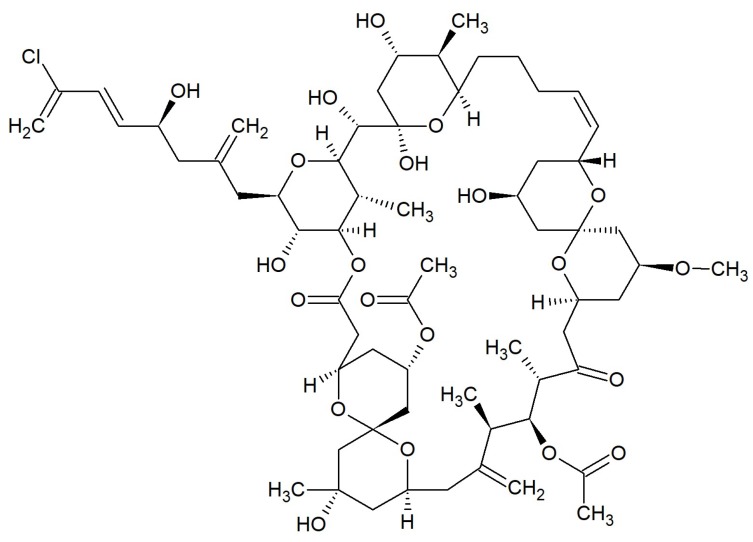
Chemical structure of 42-membered macrolide: Spongistatin 1 [100].

The spongistatins are macrocyclic lactone polyethers isolated from marine porifera. Spongistatin 1 (Figure 12) was discovered in an Indian Ocean *Spongia* species [100] and *Hyrtios erecta* together with spongistatins 2 and 3 [101]. Spongistatins 4–7 were obtained from the southeast African *Spirastrella spinispirulifera* [102,103]. All of these antibiotics inhibited the growth of *Candida albicans* and *Cryptococcus neoformans* in disk diffusion assays. Furthermore, Spongistatin 1 acted against *Issatchenkia orientalis*, *Rhodotorula mucilaginosa*, *Aspergillus fumigatus*, and *Rhizopus oligosporus* with MICs of 0.195–12.5 µg/mL [104].

In Table 1 has been presented the general characteristic of marine macrolides described in this review.

## 3. Conclusions

Marine organisms produce 34 groups of macrolides with antibacterial and/or antifungal activities. Among seventy-six antibiotics or their analog sets summarized in the Table, 36 are produced by bacteria, 18 by fungi, ten by sponges, seven by algae, two by porifera and one by nudibranch. At the same time, 29 macrolides or their groups have antifungal activity, 25 have antibacterial, and 20 have both antifungal and antibacterial. Summarizing, marine organisms are abundant in natural macrolides, which may be used in the future for the treatment of bacterial and fungal infections. Marine macrolides can also be potential drugs applicable against pathogens resistant to currently known antibiotics, which is also presented in other papers [105,106,107].

## Figures and Tables

**Table 1:** The general characteristic of marine macrolides having antimicrobial activity.

No.	Macrolide	Source	Target	References
1	(19Z)-halichondramide	sponge *Chondrosia corticata*	Fungi: *Candida albicans*, *Aspergillus niger, Aspergillus fumigatus*, *Trichophyton rubrum*, *T. mentagrophytes*	[75,76]
2	(S)-dehydrocurvularin	fungi *Curvularia* sp.	Fungi: *Phytophthora capsici*	[21]
3	11-hydroxycurvularin	actinomycete *Pseudonocardia* sp.	Bacteria: *Escherichia coli*	[24]
4	13-Deoxytedanolide	sponge *Mycale adhaerens*	Fungi: *Saccharomyces cerevisiae*	[59]
5	15G256w	fungus *Hypoxylon oceanicum*	Fungi: *Neurospora crassa*	[60,61]
6	15G256ι	fungus *Hypoxylon oceanicum*	Fungi: *Neurospora crassa*	[60,61]
7	19-*O*-demethylscytophycin C	algae *Scytonema mirabile*, *S. burmanicum*, *S. ocellatum*	Fungi: *Aspergillus oryzae*, *Candida albicans*, *Penicillium notatum*, *Saccharomyces cerevisiae*	[68]
8	5-hydroxy-de-*O*-methyllasiodiplodin	fungus No. ZZF36	Bacteria: *Staphylococcus aureus*	[38]
9	6-hydroxyscytophycin B	algae *Cylindrospermum muscicola, Scytonema mirabile*, *S. burmanicum*, *S. ocellatum*	Fungi: *Aspergillus oryzae*, *Candida albicans*, *Penicillium notatum*, *Saccharomyces cerevisiae*	[67,68]
10	7-*O*-malonylmacrolactin A	bacteria *Bacillus subtilis*	Bacteria: *Staphylococcus aureus, Enterococcus* sp., *Burkholderia cepacia;* Fungi: *Candida crusei*	[79,80]
11	7-*O*-succinylmacrolactin A and F	bacteria *Bacillus* sp.	Bacteria: *Bacillus subtilis, Staphylococcus aureus*	[78]
12	8’-hydroxyzearalanone	fungi *Penicillium* sp.	Fungi: *Pyricularia oryzae*	[45,47]
13	Amantelide A	cyanobacterium from family Oscillatoriales	Bacteria: *Staphylococcus aureus*, *Pseudomonas aeruginosa;* Fungi: *Dendryphiella salina*, *Lindra thalassiae*, *Fusarium* sp.	[99]
14	Amantelide B	cyanobacterium from family Oscillatoriales	Fungi: *Dendryphiella salina*	[99]
15	Amphidinolide Q	dinoflagellate *Amphidinium* sp.	Bacteria: *Staphylococcus aureus*, *Bacillus subtilis*, *Escherichia coli;* Fungi: *Candida albicans*	[35]
16	Aryl-crowned polyketide macrolactin	bacterium *Bacillus subtilis*	Bacteria: *Escherichia coli*, *Aeromonas hydrophilla*, *Pseudomonas aeruginosa, Klebsiella pneumoniae, Vibrio* sp.	[87]
17	Azalomycin F analogs	bacteria *Streptomyces* sp.	Bacteria: *Staphylococcus aureus*, *Bacillus subtilis, Escherichia coli;* Fungi: *Candida albicans*	[94,95]
18	Bahamaolide A	actinomycete *Streptomyces* sp.	Fungi: *Candida albicans, Aspergillus fumigatus*, *Trichophyton rubrum*, *T. mentagrophytes*	[95]
19	Borrelidin	actinomycete *Nocardiopsis* sp.	Bacteria: *Enterococcus faecalis*, *E. faecium*, *Proteus hauseri*, *Klebsiella pneumoniae*, *Salmonella enterica*	[53]
20	Borrelidins C and D	actinomycete *Nocardiopsis* sp.	Bacteria: *Salmonella enterica*	[55]
21	Bromophycolides P and Q	alga *Callophycus serratus*	Bacteria: *Staphylococcus aureus, Enterococcus faecium*	[50]
22	Butremycin	bacteria *Micromonospora* sp.	Bacteria: *Staphylococcus aureus; Escherichia coli*	[49]
23	Chalcomycins A and B	bacteria *Streptomyces* sp.	Bacteria: *Staphylococcus aureus, Bacillus subtilis, Escherichia coli*	[51,52]
24	Curvularin	fungi *Curvularia* sp., *Eupenicillium* sp.	Bacteria: *Bacillus subtilis;* Fungi: *Phytophthora capsici, Saccharomyces cerevisiae, Sclerotinia sclerotiorum*	[21,25]
25	Dendrodolides A, C and M	fungi *Cladosporium* sp.	Bacteria: *Bacillus cereus*, *Tetragenococcus halophilus*, *Staphylococcus epidermidis*, *Staphylococcus aureus*, *Escherichia coli*, *Pseudomonas putida*, *Nocardia brasiliensis*, *Vibrio parahaemolyticus*	[36]
26	de-*O*-methyllasiodiplodin	fungus No. ZZF36	Bacteria: *Staphylococcus aureus, Bacillus subtilis*, *Salmonella enteritidis;* Fungi: *Candida albicans*, *Fusarium oxysporum* f.sp. *cubense*	[38]
27	Dihydrochalcomycin	bacteria *Streptomyces* sp.	Bacteria: *Staphylococcus aureus*	[52]
28	Dihydrohalichondramide	sponge *Halichondria* sp.	Fungi: *Candida albicans*	[74]
29	Gageomacrolactins	bacterium *Bacillus subtilis*	Bacteria: *Staphylococcus aureus*, *Bacillus subtilis*, *B. cereus, Escherichia coli*, *Salmonella typhi*, *Pseudomonas aeruginosa;* Fungi: *Aspergillus niger*, *Botrytis cinerea*, *Colletotrichum acutatum*, *Candida albicans*, *Rhizoctonia solani*	[71]
30	Halichondramide	sponge *Halichondria* sp.	Fungi: *Candida albicans, Trichophyton mentagrophytes*, *Aspergillus fumigatus*, *Trichophyton rubrum*, *T. mentagrophytes*	[73,76]
31	Isohalichondramide	sponge *Halichondria* sp.	Fungi: *Candida albicans*	[74]
32	Kabiramide C	unidentified nudibranch	Fungi: *Candida albicans*, *Aspergillus niger*, *Penicillium citrium*, *Trichophyton interdigitae*	[64]
33	Lasiodiplodin	fungus No. ZZF36	Bacteria: *Staphylococcus aureus, Bacillus subtilis;* Fungi: *Fusarium oxysporum*	[38]
34	Leucascandrolide A	sponge *Leucascandra caveolata*	Fungi: *Fusarium oxysporum*, *Helminthosporium sativum*, *Phytophtora hevea*, *Botrytis cinerea*, *Pyricularia oryzae*, *Candida albicans*	[57]
35	Lobophorin A	bacteria actinomycete	Bacteria: *Bacillus thuringensis*	[41,43]
36	Lobophorin B	bacteria actinomycete	Bacteria: *Bacillus thuringensis, Bacillus subtilis*	[41,44]
37	Lobophorin E	bacteria *Streptomyces* sp.	Bacteria: *Bacillus thuringensis, Bacillus subtilis*	[43,44]
38	Lobophorin F	bacteria *Streptomyces* sp.	Bacteria: *Bacillus thuringensis, Bacillus subtilis*, *Staphylococcus aureus, Enterococcus faecalis*	[43,44]
39	Lobophorin H	bacteria *Streptomyces* sp.	Bacteria: *Bacillus subtilis, Staphylococcus aureus*	[44]
40	Lobophorin I	bacteria *Streptomyces* sp.	Bacteria: *Bacillus subtilis*	[44]
41	Macrolactin A	bacteria *Bacillus* sp., *B. subtilis, B. marinus*	Bacteria: *Staphylococcus aureus*, *Bacillus subtilis, Escherichia coli;* Fungi: *Aspergillus niger*, *Botrytis cinerea*, *Colletotrichum acutatum*, *Candida albicans*, *Rhizoctonia solani*	[71,77,82,83]
42	Macrolactin B	bacteria *Bacillus subtilis, B. marinus*	Bacteria: *Staphylococcus aureus*, *Escherichia coli*; Fungi: *Aspergillus niger*, *Botrytis cinerea*, *Colletotrichum acutatum*, *Candida albicans*, *Rhizoctonia solani, Pyricularia oryzae*, *Alternaria solani*	[71,82,83]
43	Macrolactin D	bacterium *Bacillus marinus*	Bacteria: *Staphylococcus aureus;* Fungi: *Pyricularia oryzae, Alternaria solani*	[83]
44	Macrolactin F	bacteria *Bacillus* sp., *B. subtilis*	Bacteria: *Staphylococcus aureus*, *Bacillus subtilis;* Fungi: *Aspergillus niger*, *Botrytis cinerea*, *Colletotrichum acutatum*, *Candida albicans*, and *Rhizoctonia solani*	[71,77,78]
45	Macrolactin K	bacteria *Bacillus* sp.	Bacteria: *Staphylococcus aureus*, *Bacillus subtilis*	[77]
46	Macrolactin N	bacteria *Bacillus subtilis*	Bacteria: *Escherichia coli, Staphylococcus aureus*, *Bacillus subtilis*	[81]
47	Macrolactin S	bacteria *Bacillus* sp., *B. marinus, B. amyloliquefaciens*	Bacteria: *Escherichia coli*, *Bacillus subtilis, Staphylococcus aureus*	[82,83,84]
48	Macrolactin V	bacterium *Bacillus amyloliquefaciens*	Bacteria: *Escherichia coli, Bacillus subtilis, Staphyloccocus aureus*	[84]
49	Macrolactin W	bacteria *Bacillus* sp., *B. subtilis*	Bacteria: *Bacillus subtilis*, *Staphylococcus aureus*, *Escherichia coli, Pseudomonas aeruginosa;* Fungi: *Aspergillus niger*, *Botrytis cinerea*, *Colletotrichum acutatum*, *Candida albicans*, *Rhizoctonia solani*	[71,85]
50	Macrolactins G, H, I, J, L and M	bacteria *Bacillus* sp.	Bacteria: *Staphylococcus aureus*, *Bacillus subtilis*	[77]
51	Maduralide	bacteria actinomycete	Bacteria: *Bacillus subtilis*	[88]
52	Marinisporolides A and B	actinomycete *Marinispora* sp.	Fungi: *Candida albicans*	[93]
53	Misakinolide A	sponge *Theonella* sp.	Fungi: *Candida albicans*	[62]
54	Modiolide A	fungi *Paraphaeosphaeria* sp., *Curvularia* sp.	Bacteria: *Micrococcus luteus, Staphylococcus aureus;* Fungi: *Neurospora crassa, Phytophthora capsici, Microsporum gypseum*	[21,26,27]
55	Modiolide B	fungi *Paraphaeosphaeria* sp.	Bacteria: *Micrococcus luteus;* Fungi: *Neurospora crassa*	[27]
56	Neohalichondramide	sponge *Chondrosia corticata*	Bacteria: Fungi: *Candida albicans*, *Aspergillus niger*	[75]
57	Neomaclafungins A-I	bacteria *Actinoalloteichus* sp.	Fungi: *Trichophyton mentagrophytes*	[89]
58	Neurymenolide A	alga *Neurymenia fraxinifolia*	Bacteria: *Staphylococcus aureus*, *Enterococcus faecium*	[53]
59	Phomolide A	fungi *Phomopsis* sp.	Bacteria: *Escherichia coli;* Fungi: *Candida albicans*, *Saccharomyces cerevisiae*	[28]
60	Phomolide B	fungi *Phomopsis* sp.	Bacteria: *Escherichia coli;* Fungi: *Candida albicans*, *Saccharomyces cerevisiae*	[28]
61	Phorboxazoles A and B	sponge *Phorbas* sp.	Fungi: *Candida albicans*, *Saccharomyces carlsbergensis*	[90]
62	PM100117	bacterium *Streptomyces caniferus*	Fungi: *Candida albicans, Saccharomyces cerevisiae*	[97,98]
63	PM100118	bacterium *Streptomyces caniferus*	Fungi: *Candida albicans*	[97]
64	Reedsmycins A-E	bacteria *Streptomyces* sp., *S. youssoufiensis*	Fungi: *Candida albicans*	[91,92]
65	Scytophycins	algae *Scytonema* sp., *S. pseudohofmanni, Cylindrospermum muscicola, Anabaena* sp., *Nostoc* sp.	Fungi: *Candida albicans*, *Aspergillus flavus*	[66,67,71]
66	Secohalichondramide	sponge *Chondrosia corticata*	Fungi: *Candida albicans*, *Aspergillus niger*	[75]
67	Spongistatin 1	porifera *Spongia* sp., *Hyrtios erecta*	Fungi: *Candida albicans*, *Cryptococcus neoformans, Issatchenkia orientalis*, *Rhodotorula mucilaginosa*, *Aspergillus fumigatus*, *Rhizopus oligosporus*	[100,101,104]
68	Spongistatins 2-7	porifera *Hyrtios erecta, Spirastrella spinispirulifera*	Fungi: *Candida albicans*, *Cryptococcus neoformans*	[101,102,103,104]
69	Sporiolide A	fungi *Cladosporium* sp.	Bacteria: *Micrococcus luteus;* Fungi: *Aspergillus niger*, *Candida albicans*, *Cryptococcus neoformans*, *Neurospora crassa*	[39]
70	Sporiolide B	fungi *Cladosporium* sp.	Bacteria: *Micrococcus luteus*	[39]
71	Tolytoxin (6-hydroxy-7-*O*-methylscytophycin B)	algae *Cylindrospermum muscicola, Scytonema mirabile*, *S. burmanicum*, *S. ocellatum, Tolypothrix conglutinata* var. *colorata*	Fungi: *Aspergillus oryzae*, *Candida albicans*, *Penicillium notatum*, *Saccharomyces cerevisiae Alternaria alternata*, *Bipolaris incurvata*, *Calonectria critalarae*, *Colletotrichum coccodes*, *Phyllosticta capitalensis*, *Phytophtora nicotianae*, *Rhizoctonia solani*, *Sclerotium rofsii*, *Thielaviopsis paradoxa*. *Trichophyton mentagrophytes*	[67,68,69]
72	Xestodecalactone B	fungus *Penicillium* cf. *montanense*	Fungi: *Candida albicans*	[32]
73	Zearalanone	fungi *Penicillium* sp., *Fusarium* sp.	Bacteria: *Staphylococcus aureus;* Fungi: *Pyricularia oryzae Cryptococcus neoformans*	[45,47,48]
74	αβ-dehydrocurvularin	fungi *Eupenicillium* sp.	Bacteria: *Bacillus subtilis, Staphylococcus aureus;* Fungi: *Saccharomyces cerevisiae, Sclerotinia sclerotiorum*	[25]
